# Attenuating mitochondrial dysfunction-derived reactive oxygen species and reducing inflammation: the potential of Daphnetin in the viral pneumonia crisis

**DOI:** 10.3389/fphar.2024.1477680

**Published:** 2024-10-18

**Authors:** Yuan Yuan, Runyuan Li, Yinji Zhang, Yuanxin Zhao, Qingqing Liu, Jian Wang, Xiaoyu Yan, Jing Su

**Affiliations:** ^1^ Key Laboratory of Pathobiology, Department of Pathophysiology, Ministry of Education, College of Basic Medical Sciences, Jilin University, Changchun, China; ^2^ Jilin Province Xidian Pharmaceutical Sci-Tech Development Co.,Ltd, Panshi, Jilin, China

**Keywords:** Daphnetin, inflammation, antioxidant, mitochondrial, oxidationreduction regulation

## Abstract

Amidst the global burden of viral pneumonia, mitigating the excessive inflammatory response induced by viral pneumonia has emerged as a significant challenge. Pneumovirus infections can lead to the persistent activation of M1 macrophages, culminating in cytokine storms that exacerbate pulmonary inflammation and contribute to the development of pulmonary fibrosis. Mitochondria, beyond their role as cellular powerhouses, are pivotal in integrating inflammatory signals and regulating macrophage polarization. Mitochondrial damage in alveolar macrophages is postulated to trigger excessive release of reactive oxygen species (ROS), thereby amplifying macrophage-mediated inflammatory pathways. Recent investigations have highlighted the anti-inflammatory potential of Daphnetin, particularly in the context of cardiovascular and renal disorders. This review elucidates the mechanisms by which viral infection-induced mitochondrial damage promotes ROS generation, leading to the phenotypic shift of alveolar macrophages towards a pro-inflammatory state. Furthermore, we propose a mechanism whereby Daphnetin attenuates inflammatory signaling by inhibiting excessive release of mitochondrial ROS, thus offering mitochondrial protection. Daphnetin may represent a promising pharmacological intervention for viral pneumonia and could play a crucial role in addressing future pandemics.

## 1 Introduction

The emergence of Severe Acute Respiratory Syndrome Coronavirus 2 (SARS-CoV-2) since 2019 has precipitated a global public health crisis (Saleh et al., 2020). As of 21 July 2024, over 775 million confirmed cases and more than seven million deaths have been reported globally since the beginning of pandemic (World Health, 2024). Coronavirus disease 2019 (COVID-19) has been reported to have a higher mortality rate for infected males than females (Gausman and Langer, 2020; Gebhard et al., 2020), with evidence from China suggesting a mortality rate of 2.8% for males and 1.7% for females. Further, sex-disaggregated data for COVID-19 in several European countries show a similar number of cases between the sexes, but more severe outcomes in aged men (Gebhard et al., 2020). The pathophysiology of SARS-CoV-2 infection involves significant airway damage driven by an excessive inflammatory response, which exacerbates airway injury (Tang et al., 2020). This form of viral pneumonia presents with a spectrum of respiratory symptoms that vary based on the patient’s demographics and medical history, including sore throat, cough, fever, chills, headache, shortness of breath, dyspnea, myalgia, and anosmia or ageusia (Huang et al., 2020; Li et al., 2020). Claudia Ravaglia and colleagues conducted morphological and molecular characterizations of transbronchial lung cryo-biopsies (TBLC) in patients with persistent lung disease post-SARS-CoV-2 infection, revealing systemic symptoms such as fever and fatigue, along with persistent ground-glass opacities, peribronchial patterns, and solid changes on computerized tomography (CT) imaging (Ravaglia et al., 2022). Patients with severe viral pneumonia can experience acute respiratory distress syndrome (ARDS), which can rapidly progress to respiratory failure and mortality.

Severe viral pneumonia is typified by persistent pulmonary inflammation, elevated levels of inflammatory cytokines, viral RNA presence, and a sustained interferon (IFN) response (Sefik et al., 2022). There is increasing evidence that the rapid progression of disease in patients with viral pneumonia is linked to an excessive inflammatory state known as a “cytokine storm” (Saleh et al., 2020; Jiang et al., 2022) and upregulates the expression of related proinflammatory genes (Table 1). The continuous activation of macrophages of the M1 phenotype leads to the persistent production of inflammatory mediators. This uncontrolled inflammation disrupts the subepithelial basement membrane, damages and detaches endothelial cells of small blood vessels, and persistently activates fibroblasts, ultimately resulting in fibrotic changes and compromised alveolar function. Alveolar epithelial cells (AECs), which are the primary targets of the influenza virus, exhibit a high infection rate that is associated with apoptotic death (Lindley et al., 2016). Severe viral infections can damage AECs, compromise barrier integrity, and initiate repeated epithelial injuries, which are pivotal in the development of pulmonary fibrosis (Korfei et al., 2008). Compared to other etiologies of pulmonary fibrosis, post-viral infection pulmonary fibrosis progresses more rapidly, is more severe, and is more restrictive. Therefore, significant attention has been directed towards inhibiting the inflammatory response driven by the sustained activation of M1 macrophages.

**TABLE 1:** Inflammation-related genes upregulated in viral pneumonia.

Gene name	Related pathways	Role in viral pneumonia
TNF	MIF; TNFR1 NF-κB	It is potent pyrogen causing fever by direct action or by stimulation of interleukin-1 secretion and is implicated in the induction of cachexia, Under certain conditions it can stimulate cell proliferation and induce cell differentiation
IL-6	STAT3; NF-κB; MAPK	Rapid production of IL-6 contributes to host defense during infection and tissue injury, but excessive IL-6 synthesis is involved in disease pathology. In the innate immune response, upon recognition of pathogens through toll-like receptors (TLRs) at the site of infection or tissue injury (Probable)
IL-10	MIF; TGF-β	It downregulates the expression of Th1 cytokines, MHC class II Ags, and costimulatory molecules on macrophages. It also enhances B cell survival, proliferation, and antibody production. This cytokine can block NF-kappa B activity, and is involved in the regulation of the JAK-STAT signaling pathway
IL-1β	Nrf2; MIF	This cytokine is an important mediator of the inflammatory response, and is involved in a variety of cellular activities, including cell proliferation, differentiation, and apoptosis
TLR4	TICAM1-TBK1-IRF3; NLRP3 NF-κB	The protein encoded by this gene is a member of the Toll-like receptor (TLR) family which plays a fundamental role in pathogen recognition and activation of innate immunity
CXCL8	MIF; TGF-β	The protein is a major mediator of the inflammatory response. Viral products rapidly induce IL-8 expression. IL-8 also participates with other cytokines in the proinflammatory signaling cascade and plays a role in systemic inflammatory response syndrome (SIRS)

Mitochondrial metabolism plays a crucial role in regulating macrophage polarization and immune adaptation (Mills and O’Neill, 2016). Viral-induced mitochondrial dysfunction leads to damage in the electron transport chain (ETC.) and a reorganization of energy metabolism, characterized by an increase in aerobic glycolysis, a reduction in ATP synthesis, and elevated production of mitochondrial reactive oxygen species (mtROS). These conditions favor the activation of pro-inflammatory M1 macrophages. Studies have demonstrated that mitochondrial dysfunction induced by viral or bacterial infections can hinder the transition of M1 macrophages to the anti-inflammatory M2 phenotype (Van den Bossche et al., 2016). This persistent activation of M1 macrophages exacerbates the inflammatory response (Kelley et al., 2019), leading to tissue damage and pathological inflammation.

Pathogens persist within host cells by manipulating mitochondrial function, resulting in increased mtROS production. Research by Prasada Kabekkodu et al. indicates that SARS-CoV manipulates immune mechanisms to survive and replicate within host cells. The infection disrupts cellular metabolism, favoring viral replication and leading to, ETC., disruption and mitochondrial dysfunction, which in turn promotes chronic inflammation. SARS-CoV proteins have been shown to decrease mitochondrial membrane potential (MMP), increase mtROS levels, and alter mitochondrial mass. These changes further contribute to the inflammatory state and pathogen persistence (Prasada Kabekkodu et al., 2023).

mtROS and their associated inflammatory signaling pathways significantly contribute to the polarization of macrophages towards the M1 phenotype. Upon SARS-CoV-2 entry into the respiratory tract and subsequent replication, the antiviral immune response is initiated, with mtROS playing a critical role in the immune functions of macrophages and dendritic cells (Szczesny-Malysiak et al., 2020). As the viral infection persists, mtROS stimulate the production of additional pro-inflammatory cytokines (Li et al., 2013), which activate various inflammatory signaling pathways, culminating in a “cytokine storm”. This dysregulated inflammatory response further exacerbates mitochondrial dysfunction and increases mtROS production. Persistent activation of M1 macrophages leads to oxidative stress and host cell damage, impairing lung function and oxygen exchange (Georgieva et al., 2023), and potentially resulting in tissue damage and multi-organ failure (de Las Heras et al., 2020; Muhammad et al., 2021), including the progression to ARDS and respiratory failure (Borges et al., 2021).

Currently, the conventional treatments for viral pneumonia include hormonal drugs, antiviral drugs, etc (Jiang et al., 2022). The former has big side effects and may have problems such as immune system suppression, bone density reduction, edema, and blood glucose increase; the latter is difficult to develop and has a long research and development period and is ineffective in the case of rapid virus mutation (Yuan et al., 2023). In addition, vaccines, as one of the measures to control viral pneumonia, played an important role in the COVID-19 pandemic, but they are mainly for prevention and control and have difficulties in popularization and public acceptance (Zhang et al., 2023).

Coumarins are aromatic compounds with a benzene and α-pyranone structure, widely found in various plants, and to a lesser extent in some fungi and bacteria. These compounds are secondary metabolites present in different parts of plants, such as roots, seeds, nuts, flowers, and fruits, with high concentrations in families such as Umbelliferae. Compositae, and Rosaceae. Coumarins play a significant role in human health due to their biological activities. Daphnetin, a specific type of coumarin, has demonstrated notable therapeutic effects in anti-inflammatory and antioxidant capacities, particularly in cardiovascular and renal diseases. The mechanisms of Daphnetin are closely linked to mitigating mitochondrial dysfunction, reducing ROS production, and modulating energy metabolism, suggesting its potential role in the treatment of viral pneumonia. This review synthesizes current knowledge on the anti-inflammatory and antioxidant properties of coumarin analogs like Daphnetin, proposing that Daphnetin may balance intracellular redox reactions to mitigate oxidative damage by regulating mtROS and limiting macrophage-mediated inflammatory signaling by improving mitochondrial function. This could pave the way for new pharmacological strategies in treating viral pneumonia.

## 2 Macrophage phenotypic remodeling in pneumonia progression

The initiation of a systemic inflammatory response is characterized by the hyperactivation of immune cells and the extensive release of cytokines (Yan et al., 2024). Macrophages are critical players in the pathogenesis of inflammatory lung diseases, contributing to the onset and progression of inflammation through the secretion of cytokines, chemokines, and transcription factors. They are pivotal in driving the cytokine storm observed in severe cases. In the lung microenvironment, macrophages predominantly exist as alveolar macrophages (AM) and interstitial macrophages (IM) (Saradna et al., 2018). These cells exhibit remarkable plasticity and can alter their phenotype in response to diverse environmental stimuli (Murray et al., 2014). AMs can differentiate into either M1 or M2 macrophages, where M1 macrophages are primarily involved in pro-inflammatory responses for host defense, and M2 macrophages facilitate anti-inflammatory responses and tissue remodeling (Liu et al., 2014).

The polarization of AMs to the M1 phenotype triggers the exudative phase of inflammation, and a dysregulated immune response can mediate lung injury in severe viral infections (Higgins et al., 2008). During viral infections, innate immune cells, particularly macrophages, serve as the frontline defense. The activation of pattern recognition receptors (PRRs) and a pro-inflammatory microenvironment drive macrophages towards a pro-inflammatory M1 phenotype (Gauthier and Chen, 2022). Nucleic acid receptors located on alveolar epithelium and macrophages are activated, upregulating PRR expression and enhancing the downstream activity of nuclear factor kappa B (NF-κB). M1 macrophages subsequently produce inflammatory cytokines, chemokines, and toxic molecules that recruit additional inflammatory cells such as monocytes and neutrophils. Key inflammatory mediators include tumor necrosis factor-alpha (TNF-α), interleukin-1 beta (IL-1β), IL-6, monocyte chemoattractant protein-1 (MCP-1), and ROS. TNF-α and IL-1 family members amplify inflammatory signaling, prolonging or exacerbating the intrinsic immune response. Notably, overexpression of IL-6 is strongly correlated with the severity of the cytokine storm (Sefik et al., 2022). Studies have shown that the progression of COVID-19 to severe illness is associated with significant dysregulation of inflammatory factors, with cytokine storms causing extensive damage, leading to ARDS and even mortality in COVID-19 patients (Mehta et al., 2020). During acute SARS-CoV-2 infection, the monocyte and macrophage systems undergo substantial remodeling, marked by increased infiltration of inflammatory monocytes and a highly pro-inflammatory macrophage phenotype in critically ill patients (Merad and Martin, 2020; Liao et al., 2020).

## 3 Mitochondrial function as a key determinant of macrophage polarization

Mitochondria, beyond serving as cellular powerhouses, are crucial sources of dynamic signaling in response to environmental stimuli (Mills and O’Neill, 2016). The metabolic phenotype of macrophages is intricately linked to mitochondrial function, with precise regulation essential for disease control and maintenance of tissue homeostasis (Van den Bossche et al., 2017). Studies indicate that alterations in mitochondrial metabolism and physiology are fundamental in determining macrophage activation states, including changes in oxidative metabolism, mitochondrial membrane potential, the tricarboxylic acid (TCA) cycle, and the release of mtROS and mitochondrial DNA (mtDNA) (Wang et al., 2021). When viral or bacterial infections induce mitochondrial dysfunction, the resultant damage to the electron transport chain leads to a metabolic shift from oxidative phosphorylation (OXPHOS) to aerobic glycolysis, similar to the “Warburg effect”. This metabolic reprogramming upregulates the expression of pro-inflammatory genes in immune cells (Schulze and Harris, 2012) and generates intermediate metabolites that promote the polarization of macrophages towards the M1 phenotype (Kelly and O’Neill, 2015). This metabolic remodeling, critical for regulating the inflammatory state of macrophages, stimulates reverse electron transport (RET), generating mtROS through increased succinate oxidation and inhibited ATP synthesis (Skulachev et al., 2023). This process sustains the inflammatory M1 phenotype and prevents the transition to the anti-inflammatory M2 phenotype (O’Neill, 2016). Consequently, targeting mitochondrial function and inhibiting the dysfunctional respiratory chain to reduce excessive mtROS production may prevent the prolonged activation of M1 macrophages. This approach presents a potential therapeutic strategy for managing viral-induced inflammatory diseases.

## 4 Mitochondrial-generated ROS and macrophage-mediated pro-inflammatory signaling

### 4.1 The crucial role of mtROS in antiviral immune defense

Recent research has elucidated that mtROS production is actively stimulated by various signals, including pathogen-associated molecular patterns (PAMPs), damage-associated molecular patterns (DAMPs), and endogenous molecules such as TNF (Murphy and Hartley, 2018). These signals alert cells to disruptions in mitochondrial homeostasis and activate pertinent immune responses. Upon entry into the respiratory tract, SARS-CoV-2 begins replication, and its single-stranded RNA is recognized by retinoic acid-inducible gene I (RIG-I)-like receptors in alveolar macrophages. This recognition activates the mitochondrial antiviral signaling protein (MAVS), initiating antiviral defenses (Zhang et al., 2022). Wu et al. reported that MAVS mediates NF-κB and type I interferon signaling during viral infection and is necessary for activating the NLR family pyrin domain containing 3 (NLRP3) inflammasome, which further triggers immune responses (Wu et al., 2023). Additionally, mitochondrial proteins such as gC1qR and NLRX1 (or NOD9) directly interact with the RIG-I/MAVS pathway, modulating OXPHOS activity and subsequent ROS production (Fogal et al., 2010; Moore et al., 2008; Xu et al., 2009).

### 4.2 Viral manipulation of mitochondrial function to generate mtROS and exacerbate inflammation

Viruses manipulate mitochondrial function to produce mtROS, primarily to limit their proliferation. However, mtROS also serve as signals for various cellular pathways. Viruses can induce or inhibit specific mitochondrial processes, thereby disrupting the balance of mtROS production and metabolism, causing oxidative stress, and exploiting cellular metabolic disturbances for replication and proliferation (Anand and Tikoo, 2013). For instance, Hu et al. reported that respiratory syncytial virus (RSV) infection leads to impaired mitochondrial respiration, loss of mitochondrial membrane potential, and increased mtROS, which facilitates viral replication in host cells (Hu et al., 2019). Similarly, studies have shown that SARS-CoV-2 disrupts mitochondrial dynamics, leading to mitochondrial dysfunction characterized by decreased MMP, calcium homeostasis imbalance, opening of the mitochondrial permeability transition pore (MPTP), and elevated ROS release (Ganji and Reddy, 2020).

**FIGURE 1:**
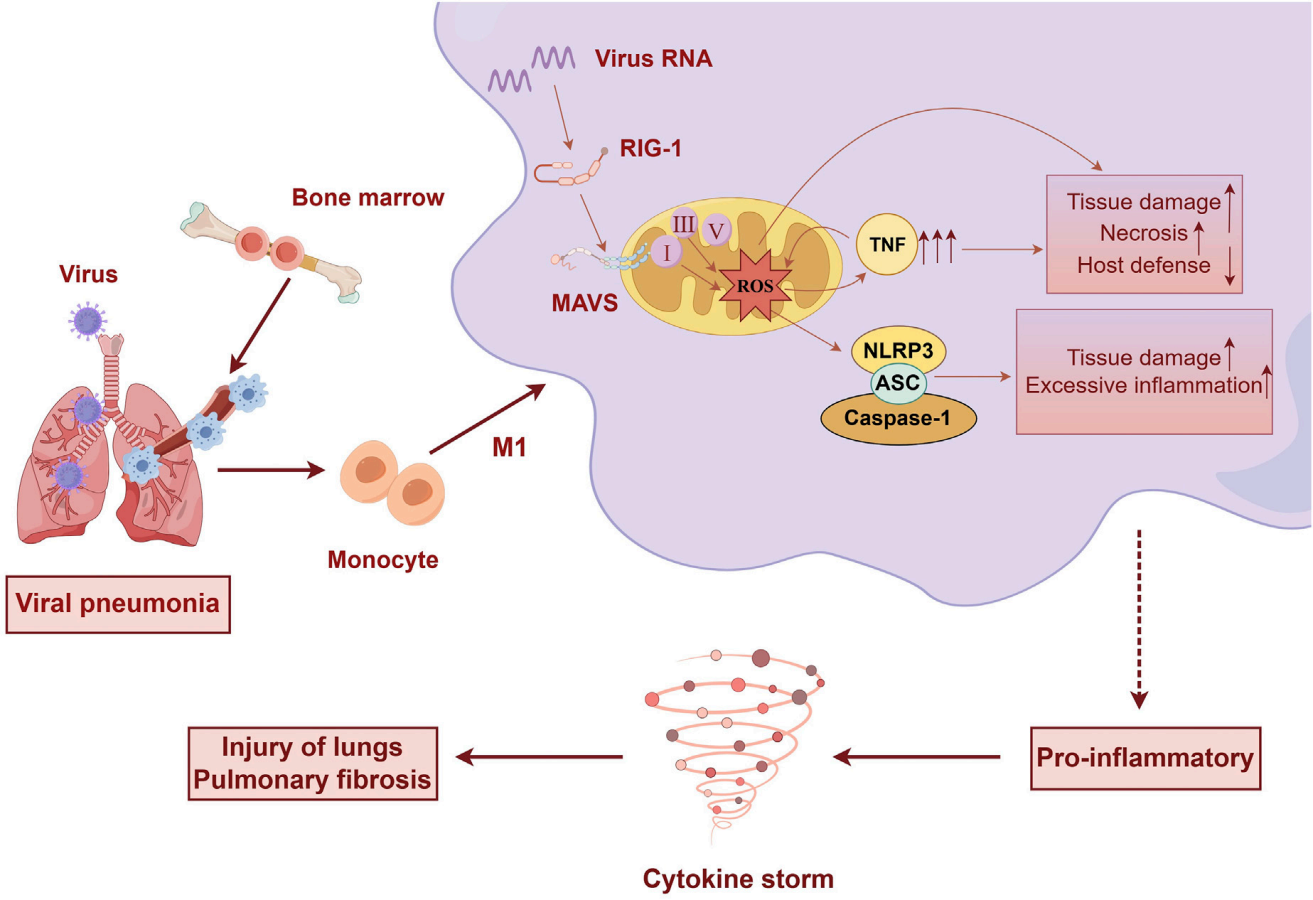
Upon viral infection of the lungs, significant alterations occur in immune cells. Bone marrow-derived monocytes differentiate into pro-inflammatory macrophages. Viral invasion activates MAVS on the mitochondria, leading to mitochondrial dysfunction, disruption of the electron transport chain, and excessive production of ROS. This cascade promotes cytokine production and the sustained activation of M1-type macrophages, culminating in a cytokine storm that causes tissue damage and lung fibrosis. (by Figdraw).

SARS-CoV-2 enters host cells, where its RNA and RNA transcripts target mitochondria, leading to disruption of the mitochondrial, ETC (Akbari and Taghizadeh-Hesary, 2023). This disruption results in the downregulation of mitochondrial respiratory chain complexes I, II, III, and V, causing mitochondrial dysfunction and increased production of ROS (Codo et al., 2020). Elevated ROS levels subsequently enhance cytokine production. Uncontrolled mtROS production precipitates an excessive inflammatory response, tissue damage, and necrosis, which are detrimental to host defense mechanisms (Silwal et al., 2020). (Figure 1)

### 4.3 Role of ROS in promoting pro-inflammatory macrophage phenotype and activating inflammatory signaling

Upon viral or bacterial invasion, M1 macrophages generate significant amounts of ROS with bactericidal properties. ROS are crucial for maintaining M1/M2 macrophage polarization and their associated functions. As key mediators in the activation of pro-inflammatory signaling, mtROS have been shown to promote the M1 phenotype by inducing cellular hydrogen peroxide production (West et al., 2011). Additionally, mitochondrial enzymes such as superoxide dismutase-2 (SOD2) and mtROS can be delivered to phagosomes containing bacteria via mitochondria-derived vesicles (MDVs). This mechanism enhances antimicrobial defense and the phagocytic activity of pro-inflammatory cells (Abuaita et al., 2018). Johan Garaude et al. demonstrated that excessive ROS production leads to the activation of Fgr in macrophages, which is linked to increased activity of mitochondrial complex II and degradation of complex I, thereby promoting pro-inflammatory macrophage polarization (Casuso and Huertas, 2021). Furthermore, mtROS act as pro-inflammatory signals, enhancing the release of pro-inflammatory mediators and sustaining the activation of M1 macrophages. This regulation occurs through the activation of relevant inflammatory signaling pathways, leading to the induction of pro-inflammatory gene expression and cytokine production.

#### 4.3.1 Regulation of inflammatory signaling pathways by mtROS

##### 4.3.1.1 NF-κB and Nrf2

Mitochondria are pivotal in the regulation of cellular inflammation, where oxidative stress-induced mitochondrial damage leads to excess production and release of ROS, modulating multiple inflammatory signaling pathways. NF-κB and Nrf2 are critical regulators of antioxidant genes and inflammatory mediators, responding to intracellular ROS changes, which suggests their roles as ROS-sensitive cellular receptors. Nrf2 is a key redox-sensitive transcriptional regulator that maintains cellular homeostasis and protects against oxidative damage by balancing redox signaling. The precise mechanisms of Nrf2 in oxidative stress and inflammatory responses remain incompletely understood. Under oxidative stress, Nrf2 dissociates from its inhibitor Keap1 and translocates to the nucleus, where it binds to antioxidant response element (ARE) sequences on DNA. This binding activates various secondary antioxidant enzymes and proteins to mitigate oxidative stress. Conversely, inhibition of Nrf2 expression can further activate inflammatory factors such as TNF-α, and thus cause a final inflammatory response. Research has demonstrated that ROS generated by mitochondrial dysfunction in corneal epithelial cells exposed to particulate matter (PM2.5) inhibit the expression of Nrf2, leading to the activation of the inflammatory protein NF-κB (p65) and downstream inflammatory cytokines IL-1β and TNF-α, ultimately resulting in inflammatory responses (Yu et al., 2023). In RGK1 cells with elevated ROS production, intracellular signaling pathways such as Nrf2, Keap1, HO-1 and -2, GCL, GST, and NQO1 are upregulated. This enhancement of signaling can be inhibited through MnSOD transfection. The findings suggest that mtROS can translocate from mitochondria to the cytosol to regulate various intracellular signals, placing them in a central position in controlling cellular signaling (Indo et al., 2023). In addition, TCBQ has been shown to increase the nuclear translocation of p65 and significantly upregulate the expression of inflammatory cytokines IL-1β, IL-6, and TNF-α by elevating ROS concentrations in PC12 cells. This process subsequently regulates the phosphorylation and activation of IκB kinase (IKK) α/β (Fu et al., 2016). The activation of NF-κB is controlled by the IKK complex, which phosphorylates IκB on Ser32 and Ser36 residues, leading to its ubiquitination and proteasome-dependent degradation (Pasparakis, 2009). Consequently, we propose that ROS activate IKK, and the activated IKK phosphorylates IκBα at serines 32 and 36. This phosphorylation results in ubiquitination and degradation of IκBα, allowing NF-κB to dissociate and translocate to the nucleus, where it regulates the expression of inflammatory mediators. Furthermore, ROS (particularly H2O2) may influence casein kinase II by modulating Syk kinase, thereby inducing tyrosine phosphorylation of IκBα, leading to its dissociation, phosphorylation, and nuclear translocation of p65 (Schoonbroodt et al., 2000; Takada et al., 2003).

##### 4.3.1.2 Inflammasome

The NLRP3 inflammasome is a critical mediator of inflammation that can detect mitochondrial dysfunction (Zhou et al., 2011). Current studies indicate that mitochondrial dysfunction, mtROS, or excessive mtDNA release may activate the NLRP3 inflammasome (Tschopp and Schroder, 2010). Stress conditions such as hypoxia or membrane damage significantly induce mtROS production. Upon detecting cellular stress, NLRP3 recruits ASC and procaspase-1, resulting in the release of pro-inflammatory cytokines IL-1β and IL-18, which drive cellular pyroptosis and inflammatory responses (Zhou et al., 2011). MnTBAP has been shown to ameliorate mitochondrial dysfunction in an albumin-induced renal tubular cell injury model by reversing mitochondrial swelling, reducing mtDNA copy number, and decreasing cytochrome c release. Additionally, MnTBAP decreases ROS production, increases ATP content, and stabilizes mitochondrial membrane potential, thereby limiting the activation of NLRP3 inflammasomes and reducing the expression of caspase-1, IL-1β, and IL-18 (Zhuang et al., 2015). In the NaAsO2-induced hepatic insulin resistance model, the inhibition of mtROS can reduce the upregulation of oxidized mitochondrial DNA (ox-mtDNA) and mitigate mitochondrial mitophagy, thereby suppressing the activation of the NLRP3 inflammasome (NLRP3, ASC, pro-caspase-1) and decreases the expression levels of caspase-1, IL-1β, and IL-18 (Jia et al., 2020).

##### 4.3.1.3 Inflammatory signaling interaction networks

The intricate network of inflammatory signaling interactions further amplifies cellular damage caused by excessive mtROS spillover. Nrf2 activation induces the expression of proteins that inhibit ROS, thereby mitigating NLRP3 inflammasome activation. Additionally, Nrf2 can directly repress the transcription of genes related to NLRP3 inflammasome, including NLRP3, proIL-1β, and proIL-1α. Activation of the NLRP3 inflammasome drives the expression of inflammatory cytokines such as IL-1, IL-6, and TNF-α, which can further stimulate the NF-κB signaling pathway through TLR4 activation. The transcription factor NF-κB is implicated in both pathways, inducing the expression of multiple inflammatory chemokines in concert with NLRP3 (Schroder and Tschopp, 2010), which is crucial for the initiation and assembly of inflammasomes. NF-κB/p65 also antagonizes the Nrf2 pathway by reducing the transcription of ARE genes, decreasing the transcriptional co-activator CBP of Nrf2, and enhancing the recruitment of HDAC3 to the ARE region (Liu et al., 2008). In P. bovis infection-induced oxidative stress in mammary epithelial cells (mMECs), characterized by increased mtROS accumulation, mitochondrial structures are severely damaged. The infection also leads to decreased Nrf2 protein expression and activation of the NF-κB/NLRP3 inflammasome pathway, resulting in an inflammatory response in MNCs. However, scavenging ROS or activating Nrf2 inhibited the NF-κB/NLRP3 inflammasome pathway, reducing the expression of p65, NLRP3, and IL-1β, and thereby mitigating the inflammatory response in infected mMECs (Zhao et al., 2022). Thus, NF-κB, Keap1/Nrf2, and NLRP3 interact and regulate intracellular oxidative stress and inflammatory responses by forming a complex, interwoven network.

In conclusion, mitochondrial dysfunction, elevated mtROS production, and dysregulated inflammatory signaling lead to the sustained activation of M1-type macrophages, commonly associated with the excessive inflammation seen in viral pneumonia. Therefore, strategies aimed at reducing mitochondrial dysfunction by decreasing mtROS production or using pharmacological interventions to modulate inflammatory signaling pathways could prevent the detrimental effects of an excessive immune response.

## 5 Daphnetin as a potential anti-inflammatory agent for viral pneumonia treatment

### 5.1 Mitochondrial protection by Daphnetin

Coumarins exhibit a broad spectrum of pharmacological activities, and recent research has highlighted the therapeutic potential of coumarin analogs in treating various diseases due to their anti-inflammatory and antioxidant properties. These effects are often achieved through the improvement of mitochondrial function, which helps attenuate oxidative stress. Studies have indicated that coumarin analogs, such as Daphnetin, may reduce oxidative damage by modulating mtROS and balancing intracellular redox reactions. Li et al. demonstrated that 5,7-Dihydroxy-4-methylcoumarin (D4M) significantly reversed the cisplatin-induced increase in MitoSOX-Red and the decrease in mitochondrial membrane potential (ΔΨm) in mouse cochlear hair cell HEI-OC1 cells. This suggests that D4M stabilized cellular mtROS levels and mitochondrial membrane potential, thereby preserving mitochondrial function (Li et al., 2023). Furthermore, Daphnetin (10 and 20 μg/mL) was shown to significantly ameliorate Ang-II-induced mtROS production and changes in mitochondrial membrane potential in H9c2 cells. It also decreased the levels of cleaved caspase-3 and the Bax/Bcl2 ratio, upregulated the nuclear translocation of Nrf2, and increased the activities of myocardial antioxidant proteins HO-1 and NQO1, as well as the endogenous antioxidant SOD. Additionally, Daphnetin reduced the lipid peroxidation marker MDA content, thereby attenuating TAC-induced oxidative damage (Syed et al., 2022).

Furthermore, coumarin analogs such as Daphnetin have demonstrated the ability to enhance mitochondrial function by inhibiting cytochrome c release and increasing the activity of the mitochondrial respiratory chain complex. In tert-butyl hydroperoxide (t-BHP)-induced RAW264.7 cells, Daphnetin (10 μg/mL) alleviated t-BHP-induced mitochondrial MMP collapse and cytochrome c release, indicating its potential in ameliorating mitochondrial dysfunction. Additional studies have shown that Daphnetin significantly inhibits ROS generation, cytochrome c release, and NLRP3 inflammasome activation, while also modulating the expression of apoptosis-related proteins Bcl-2, Bax, and caspase-3 (Lv et al., 2017). These findings provide compelling evidence for the application of Daphnetin in preventing oxidative stress-induced mitochondrial dysfunction, suggesting its potential utility in treating diseases associated with mitochondrial damage. In a mouse model of acute radiation syndrome (ARS), Beta-Escin has been shown to promote mitochondrial function by restoring cortical cytochrome c oxidase (COX) activity, a marker of brain metabolic state and mitochondrial function (Martín-Aragón et al., 2016). Similarly, in a rat model of spinal cord ischemia-reperfusion (I/R) injury, Osthole preserved mitochondrial membrane potential, reduced ROS production, increased ATP production, and inhibited cytochrome c release. Osthole also enhanced the activity of mitochondrial respiratory chain complexes I, III, and IV in spinal cord tissue (Zhou et al., 2013). The protective effect of Osthole against spinal cord I/R injury is attributed, at least in part, to its ability to preserve mitochondrial respiratory chain complex activity. Therefore, it is suggested that coumarin analogs such as Daphnetin may mitigate mitochondrial dysfunction by reversing elevated mtROS, preventing MMP collapse, inhibiting cytochrome c release, and maintaining the activity of the mitochondrial respiratory chain complex.

### 5.2 Modulation of NF-κB, Keap1/Nrf2, and NLRP3 signaling by Daphnetin

Several studies have demonstrated that both natural and synthetic coumarin analogs exhibit significant anti-inflammatory effects. Research on the anti-inflammatory properties of coumarin analogs has primarily focused on conditions such as arthritis, bronchitis, lung injury, liver injury, and kidney injury. Daphnetin (DAPH, molecular formula: C₉H₆O₄), also known as 7,8-dihydroxycoumarin, is an odorless white or off-white powder that is readily soluble in ethanol, methanol, and dimethylsulfoxide, and slightly soluble in water. Daphnetin, a simple coumarin, possesses a range of pharmacological activities, with its anti-inflammatory properties being extensively recognized in various *in vitro* and *in vivo* studies (Table 2). In a mouse model of lipopolysaccharide (LPS)-induced endotoxemia, Daphnetin (5 mg/kg in mice, 20 μM in cell) was found to reduce LPS-induced alveolar edema and inflammatory cell infiltration, decrease endotoxin lethality, and attenuate the 100 ng/mL LPS-induced inflammatory response in Raw264.7 by inhibiting the production of pro-inflammatory cytokines such as IL-1β, IL-6, and TNF-α (Shen et al., 2017). In the collagen-induced arthritis (CIA) model in rats, Daphnetin not only reduced the expression of pro-inflammatory cytokines IL-17, IL-6, and TGF-β but also inhibited the expression of Th1/Th2/Th17-type cytokines in splenic lymphocytes, demonstrating significant anti-inflammatory activity (Tu et al., 2012). Moreover, Yang S et al. demonstrated that administration of 4 mg/kg Daphnetin significantly reduced myeloperoxidase (MPO) activity in L-arginine-induced severe acute pancreatitis (SAP) mice, while markedly inhibiting the production of IL-6 and TNF-α in lung tissue. This treatment also led to a reduction in the infiltration and apoptosis of macrophages (CD11b) and neutrophils (Ly6G) within the pulmonary environment (Yang et al., 2021). These findings suggest that Daphnetin mitigates SAP-associated severe damage to alveolar structures and reduces congestion of alveolar septal capillaries.

**TABLE 2:** Dose and pharmacological activities of Daphnetin in various models.

Models/Methods	Dose	Pharmacological effects	Targets	Results stage/Administration routes	References
transverse aortic constriction-induced cardiac remodeling in mice	10 and 20 mg/kg	Antioxidant properties	Nrf2 pathway	Animal experiment/Oral	Syed et al. (2022)
NaAsO2-induced Beas-2B-cells	2.5,5 and 10 μg/mL	Antioxidant properties		Cell experiment	Lv et al. (2019)
APAP or t-BHP- induced ALF mice model	40 and 80 mg/kg	Hepatoprotective properties/Antioxidant properties		Animal experiment/Intraperitoneal injection	Lv et al. (2020)
Endotoxin-induced acute lung injury mice model	5 and 10 mg/kg	Anti-inflammation/Protective properties	NF-κB pathway	Animal experiment/Intraperitoneal injection	Yu et al. (2014)
methicillin-resistant *Staphylococcus aureus* (MRSA)- induced bacterial pneumonia mice model	10 mg/kg	Anti-inflammation/Induce autophagic response		Animal experiment/Intraperitoneal injection	Zhang et al. (2019)
A mice AAA model was established by intra-aortic infusion of porcine pancreatic elastase (PPE)	20 mg/kg	Anti-cancer properties		Animal experiment/Intraperitoneal injection	Xie et al. (2020)
LPS-induced MLE-12 cells	5,10 and 20 μg/mL	Anti-inflammation/Inhibit apoptosis and pyroptosis	NLRP3 inflammasome	Cell experiment	Guo et al. (2023)
PM2.5-CS-induced acute exacerbation of chronic obstructive pulmonary disease (AECOPD) mice model	40 mg/kg	Anti-inflammation/Inhibit pyroptosis		Animal experiment/Intraperitoneal injection	Fan et al. (2023)

#### 5.2.1 Daphnetin attenuates lung injury by limiting NF-κB activity

Recent studies have provided growing evidence that Daphnetin exhibits diverse biological activities across various models through the modulation of the NF-κB signaling pathway. Using a prototype model of LPS-induced lung inflammation and injury, Wen-wen Yu et al. (2014) demonstrated that Daphnetin (80 and 160 μM) can significantly reduce interstitial lung edema and debris deposition, thereby alleviating symptoms of lung injury. The study found that ryanodine, an inhibitor of pro-inflammatory cytokines such as IL-6, TNF-α, and IL-1β, targets TNFAIP3, selectively reverses TRAF6 ubiquitylation and inhibits the phosphorylation of IKK and IκBα to restrict NF-κB-driven pro-inflammatory signaling. Additionally, Daphnetin was shown to reduce myeloperoxidase (MPO) activity in lung tissue and block the infiltration of inflammatory cells.

In macrophages infected with *Staphylococcus aureus*, Daphnetin (160 μM) treatment led to reduced phosphorylation of p65 and its upstream signaling molecules, including IκBα and IKK, thereby diminishing the activation of the NF-κB pathway. Furthermore, it was observed that Daphnetin enhances the mTOR-dependent autophagy pathway in macrophages, which increases their bactericidal activity and offers substantial protection against *Staphylococcus* aureus-induced pneumonia (Zhang et al., 2019). This protective effect is characterized by a reduced inflammatory response, enhanced bacterial clearance, and decreased tissue damage.

#### 5.2.2 Daphnetin attenuates oxidative stress and inflammation by degrading Keap1 and activating Nrf2

In a model of NaAsO_2_-induced injury in human lung epithelial Beas-2B cells, Daphnetin (2.5, 5 or 10 μg/mL) downregulated the expression of Keap1 protein and activated Nrf2, facilitating its translocation from the cytoplasm to the nucleus. This activation of Nrf2 led to a significant attenuation of oxidative stress in arsenic-exposed human lung epithelial cells. Daphnetin also significantly upregulated the expression of Nrf2-dependent antioxidant enzymes, including GCLC, GCLM, HO-1, and NQO1. Additionally, Daphnetin enhanced the expression of the anti-apoptotic protein Bcl-2, thereby preventing arsenic-induced apoptosis in Beas-2B cells and promoting cell survival (Lv et al., 2019).

Based on the protective effects of Daphnetin against t-BHP-induced mitochondrial dysfunction, Lv et al. (Lv et al., 2020) found that Daphnetin (40 or 80 mg/kg) alleviated hepatotoxicity caused by t-BHP and acetaminophen (APAP) through modulation of the Nrf2 pathway. The pharmacological effect increased with concentration. Daphnetin was shown to reduce t-BHP-triggered hepatotoxicity and mitochondrial dysfunction in HepG2 cells, protect mice from APAP-induced acute liver failure, and extend their survival after APAP treatment. Its hepatoprotective mechanism relies on regulating the Nrf2 signaling pathway, with these benefits absent in Nrf2-deficient mice.

Thus, through the modulation of Nrf2 pathways, Daphnetin orchestrates antioxidant defense and mitochondrial homeostasis at both cellular and systemic levels.

#### 5.2.3 Daphnetin suppresses cellular pyroptosis by inhibiting NLRP3 inflammasomes

Pyroptosis is primarily triggered by the NLRP3 inflammasome in a Caspase-1 dependent manner, promoting the self-cleavage of Pro-caspase one and activating Caspase-1. Activated Caspase-1 cleaves precursors of IL-1β and IL-18 into their mature forms, thereby activating the immune system and inducing inflammation. Additionally, Caspase-1 facilitates the cleavage and polymerization of downstream GSDMD, leading to cell disintegration and perforation, which allows for the release of pyroptosis-related inflammatory cytokines IL-1β and IL-18, further amplifying the inflammatory response.

In a mouse model of LPS-induced septic lung injury, Guo et al. (2023) demonstrated that Daphnetin (5 mg/kg) inhibited the NF-κB/NLRP3 pathway through the upregulation of CD38, which led to a reduction in the excessive release of pro-inflammatory cytokines such as IL-1β, IL-18, IL-6, iNOS, and the chemokine MCP-1. Additionally, Daphnetin (5、10、20 μg/mL) mitigated the inflammatory response in MLE-12 cells by decreasing the protein expression of NLRP3, ASC, and cleaved caspase-1 to inhibit cellular pyroptosis. This indicates that Daphnetin could improve the survival rate of septic mice and alleviate lung pathological damage, including alveolar septal thickening, intrapulmonary hemorrhage, edema formation, and inflammatory cell infiltration.

Fan et al. (2023) found that in PM2.5-CS-induced acute exacerbation of chronic obstructive pulmonary disease (AECOPD) mice, Daphnetin (40 mg/kg) inhibited NLRP3 inflammasome activation, which in turn reduced caspase-1 activation and the cleavage of GSDMD. This inhibition led to a decrease in the expression of inflammatory cytokines IL-1β and IL-18, thus suppressing cellular pyroptosis. Furthermore, Daphnetin significantly reversed PM2.5-CS-induced inflammatory cell infiltration, goblet cell proliferation, mucus secretion, and lung tissue injury, thereby reducing the severity of AECOPD induced by PM2.5-CS.

**FIGURE 2:**
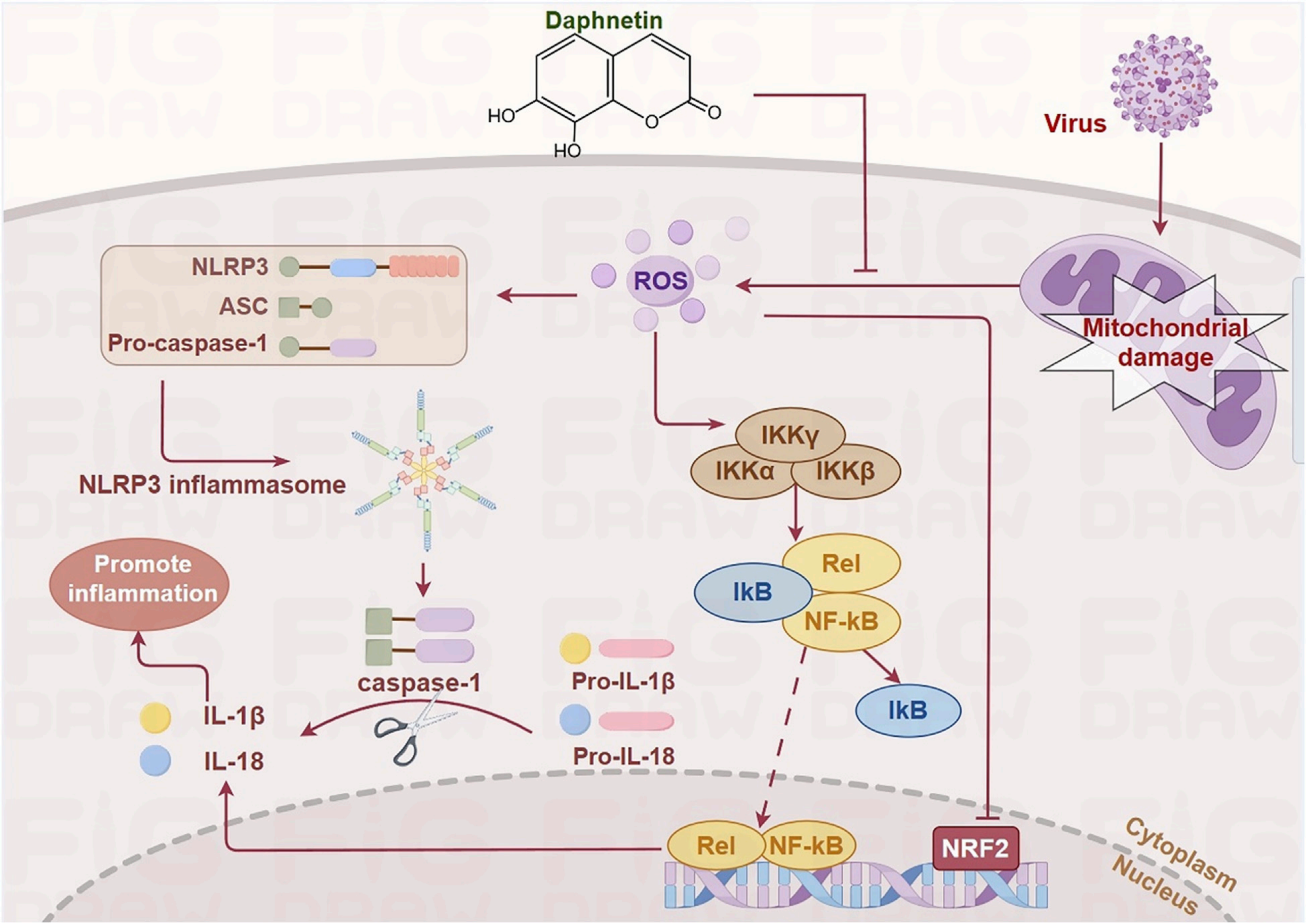
Daphnetin regulates NF-κB, Keap1/Nrf2, and NLRP3 signaling pathways by alleviating mitochondrial damage. ROS produced as a result of mitochondrial damage during viral infection activates IKK, which causes the phosphorylation and dissociation of IκB from NF-κB. This allows NF-κB to translocate into the nucleus and recruit the NLRP3 inflammasome, leading to the release of inflammatory cytokines such as IL-1β and IL-18. Additionally, the activation of these pathways inhibits the transcription of Nrf2. Daphnetin mitigates mitochondrial damage, thereby modulating these signaling pathways. (by Figdraw).

Based on the aforementioned findings, we hypothesize that Daphnetin exerts its anti-inflammatory effects by modulating the NF-κB, Keap1/Nrf2, and NLRP3 signaling pathways. This modulation ameliorates pneumonia-induced mitochondrial damage, reverses the elevation of ROS, and prevents MMP collapse, thereby mitigating the inflammatory response (Figure 2).

## 6 Discussion

Viral pneumonia is a prevalent clinical respiratory infection associated with high mortality rates. Amid the global outbreak of COVID-19, the prevention and treatment of viral pneumonia have emerged as critical public health challenges worldwide. Macrophages play a pivotal role in the pathogenesis of inflammatory lung diseases by promoting the onset and progression of inflammation through the secretion of cytokines, chemokines, and the activation of transcription factors, which collectively drive the cytokine storm. Therefore, regulating macrophage activity during pneumonia progression and balancing their pro-inflammatory and anti-inflammatory phenotypes are crucial in determining the prognosis of viral pneumonia. In this review, we hypothesize that pneumonia can induce mitochondrial dysfunction in macrophages, leading to excessive ROS release, which exacerbates inflammation and triggers a cascade of pathological reactions. Consequently, restoring mitochondrial function in macrophages is fundamental to ameliorating pneumonia. Daphnetin, a natural chemical component isolated from Daphne Korean Nakai, has demonstrated potential in improving pneumonia by mitigating mitochondrial dysfunction. It achieves this by limiting NF-κB activity, activating Nrf2, and inhibiting NLRP3 inflammasomes.

Pneumonia is an inflammatory condition of the lungs caused by various pathogens or other factors, primarily affecting the bronchial walls and alveoli. The clinical manifestations of viral pneumonia include fever, cough, wheezing, shortness of breath, dyspnea, and characteristic shadows on imaging, such as spotty or flaky opacities. Each type of viral pneumonia presents unique clinical features, with common pathogens including rhinoviruses, coronaviruses, influenza viruses, RSV, adenoviruses, and parainfluenza viruses. Despite the availability of conventional Western medications (CWM) such as antivirals, antibacterials, expectorants, and bronchodilators, there are no specific drugs that effectively control excessive inflammation. There is increasing evidence supporting the role of herbal medicine in improving lung function and reducing lung inflammation. For instance, LH has been shown to significantly inhibit SARS-CoV-2 replication, alter viral morphology, and reduce the release of cytokines such as TNF-α, IL-6, and CCL-2/MCP-1, thereby exerting anti-inflammatory effects *in vitro* (Runfeng et al., 2021). During the fight against the novel coronavirus, including Chinese herbal compounds and proprietary Chinese medicines, has been integrated with Western medicine as immune adjuvants (Liu et al., 2020; Yin et al., 2021), significantly reducing symptoms such as fever, cough, phlegm, fatigue, and chest tightness in patients.

Daphnetin is an active compound extracted from Daphne Korean Nakai. Upon absorption, Daphnetin is extensively distributed in the body, with the highest concentrations found in the kidneys, followed by the lungs, spleen, and plasma. The kidneys serve as the primary route for its excretion. Clinically, Daphnetin has been employed as an adjunctive treatment for thromboangiitis obliterans and other occlusive vascular diseases, including coronary heart disease. A clinical study involving 71 patients with thromboangiitis obliterans assessed the efficacy of Daphnetin. Patients received 300 mg three times daily for 1 month, resulting in a total effective rate of 87.3%, with significant efficacy observed at 64.8%. Furthermore, Daphnetin was found to increase skin temperature, shorten blood circulation time, enhance blood flow mapping, and extend claudication distance. Recent clinical research has shifted focus toward Zushi hemp poultice, particularly for conditions characterized by cold and damp stasis, as well as arthritis. This poultice has proven effective in alleviating pain and symptoms associated with limited joint mobility and morning stiffness in arthritis patients. It specifically addresses rheumatoid arthritis with cold-damp obstruction and knee osteoarthritis. While some patients may experience mild local skin irritations following application, these side effects are generally manageable. The LD50 of Daphnetin in mice was 5.37 g/kg. The findings from the subacute toxicity test suggested that Daphnetin exhibited no significant toxicity to the heart, liver, spleen, lungs, or kidneys of the experimental animals, demonstrating that Daphnetin did not induce any bodily harm and possesses a high safety profile. However, it is noteworthy that Daphnetin has lower bioavailability (Javed et al., 2022) and may occasionally lead to side effects such as dry mouth and hot hands.

Recent research has revealed that coumarins, including Daphnetin, also exhibit pharmacological properties such as anti-tumor and antibacterial effects. In anti-tumor applications, coumarins inhibit cancer cell proliferation by targeting telomerase, carbonic anhydrase, monocarboxylic acid transporter proteins, and modulating protein kinase activity. They downregulate oncogene expression and induce apoptosis in cancer cells (Song et al., 2020; Bhattarai et al., 2021). Research findings have shown that Daphnetin at a dosage of 20 mg/kg can significantly reduce the density of mural macrophages, T cells, and B cells in mice with abdominal aortic aneurysm (AAA) (Xie et al., 2020). These findings indicated that Daphnetin may limit the formation and progression of experimental aneurysms by inhibiting mural inflammation and angiogenesis through targeting NF-κB signaling pathways include TNF-α-induced IκBα degradation, IKK phosphorylation, and NF-κB-P65 protein translocation. For instance, a series of novel coumarin-3-carboxylic acid derivatives synthesized by Ji et al. (2021) were found to inhibit cell proliferation and induce apoptosis by lowering the expression levels of the monocarboxylic acid transporter protein MCT1, thereby inhibiting lactate transport and suppressing cellular energy metabolism. In antibacterial applications, the antimicrobial activity of coumarins is primarily due to their ability to bind to the B subunit of bacterial DNA gyrase, inhibiting DNA supercoiling by blocking ATPase activity. Coumarin analogs also regulate mitochondrial and cellular energy metabolism, which contributes to their antitumor and antibacterial effects. The diverse mechanisms of action of coumarin analogs, such as Daphnetin, are closely linked to their effects on mitochondria. Mitochondria, the organelles responsible for producing large amounts of ATP, play crucial roles in inflammatory signaling and are key in cellular changes and damage during pneumonia.

As global concerns about future pandemics continue to rise, there is an increasing focus within the scientific community on discovering specific therapeutics that can effectively treat viral pneumonia. Recent research suggests that Daphnetin possesses unique properties that may offer broad-spectrum therapeutic effects for lung inflammation caused by new viral infections. The toxicological profile of Daphnetin has been clinically validated. Based on the findings presented in this article, we hypothesize that Daphnetin can inhibit the excessive release of ROS by improving the mitochondrial function of macrophages in pneumonia. This modulation can influence the phenotypic transformation of macrophages, thereby addressing the hyperinflammatory state characteristic of pneumonia. We propose that a new therapeutic indication for Daphnetin could be developed, leveraging its potential to ameliorate the hyperinflammatory state in viral pneumonia. In addition to its potential in treating pneumonia, Daphnetin exhibits various pharmacological activities, including anti-tumor, antibacterial, and neuroprotective effects, suggesting its vast therapeutic potential and promising future prospects. However, current research on Daphnetin in the context of pneumonia is limited, with current understanding primarily based on *in vitro* studies and animal models. Further exploration into the mechanisms of Daphnetin in pneumonia is needed to provide a more comprehensive understanding. Robust preclinical and clinical trial data are crucial to validate the efficacy of Daphnetin in treating viral pneumonia in human cases. Future research efforts should concentrate on elucidating the mechanisms of action and gathering substantial evidence to support its use in clinical settings.

Overall, Daphnetin, as a compound with multiple pharmacological activities, holds significant promise for the treatment of lung inflammation caused by novel viral infections. With further research, Daphnetin is expected to play an increasingly important role in the prevention and treatment of virus-associated pneumonia.
